# Overview of the role of kinetoplastid surface carbohydrates in infection and host cell invasion: prospects for therapeutic intervention

**DOI:** 10.1017/S0031182019001355

**Published:** 2019-10-11

**Authors:** Maria Valente, Víctor M. Castillo-Acosta, Antonio E. Vidal, Dolores González-Pacanowska

**Affiliations:** Instituto de Parasitología y Biomedicina ‘López-Neyra’, Consejo Superior de Investigaciones Científicas (CSIC), Parque Tecnológico de Ciencias de la Salud, Avenida del Conocimiento, 17, 18016, Armilla, Granada, Spain

**Keywords:** Carbohydrate-binding agents, kinetoplastids, lectins, *Leishmania*, surface glycans, *Trypanosoma brucei*, *Trypanosoma cruzi*

## Abstract

Kinetoplastid parasites are responsible for serious diseases in humans and livestock such as Chagas disease and sleeping sickness (caused by *Trypanosoma cruzi* and *Trypanosoma brucei*, respectively), and the different forms of cutaneous, mucocutaneous and visceral leishmaniasis (produced by *Leishmania* spp). The limited number of antiparasitic drugs available together with the emergence of resistance underscores the need for new therapeutic agents with novel mechanisms of action. The use of agents binding to surface glycans has been recently suggested as a new approach to antitrypanosomal design and a series of peptidic and non-peptidic carbohydrate-binding agents have been identified as antiparasitics showing efficacy in animal models of sleeping sickness. Here we provide an overview of the nature of surface glycans in three kinetoplastid parasites, *T. cruzi*, *T. brucei* and *Leishmania*. Their role in virulence and host cell invasion is highlighted with the aim of identifying specific glycan–lectin interactions and carbohydrate functions that may be the target of novel carbohydrate-binding agents with therapeutic applications.

## Introduction

The occurrence of surface glycoconjugates in parasitic protozoa is of paramount importance since they are crucially involved in processes such as immune evasion, host cell invasion and endocytosis. Most parasitic protozoa undergo complex life cycles and must adapt to changing hosts and environments. Surface glycans constitute a protective barrier that contributes to adaptation and the establishment of infection by participating in the subversion of the immune response and in specific interactions with host surface molecules. Thus, specific sugar–lectin interactions are involved in the colonization of the gut of the insect vector as well as in mammalian host cell recognition and parasite internalization. In this latter process, specific glycan–lectin interactions mediate mammalian host cell recognition and parasite uptake. Pattern recognition receptors (PRRs) present on the surface of immune cells distinguish pathogen-associated molecular patterns (PAMPs). The receptors used for parasite infection vary and include complement receptors, scavenger receptors, Toll-like receptors (TLR) and mannose receptors. The understanding of these interactions will provide an insight into how protozoa implement infection and subvert the host immune response.

In relation to the immune response, several carbohydrate-binding proteins, either expressed on the surface of cells of the immune system or released, play an essential role in the control of innate and adaptive immunity. These include C-type lectin receptors, sialic acid-binding immunoglobulin (Ig)-like lectins (siglecs) and galectins that interact with distinct glycan structures (van Kooyk and Rabinovich, 2008). DC-SIGN, a C-type lectin receptor found on the surface of dendritic cells specifically binds mannose and/or fucose-terminated glycans. Siglecs are included in the group of Ig-type (I-type) lectins and interact with a wide variety of structurally distinct carbohydrate ligands (Pillai *et al*., 2012). Galectins are a family of soluble lectins that bind *β*-galactose (*β*-Gal)-containing glycoconjugates such as glycans containing *N*-acetyllactosamine and are thought to be able to associate with host membrane glycans to form a cell-surface network for an optimal receptor spacing and signalling (Liu and Rabinovich, 2005; Nieminen *et al*., 2007). While they can act as effector factors, inhibiting pathogen adhesion and entry or stimulating phagocytosis, parasites can also make use of host galectins to facilitate host cell invasion. Furthermore, in the case of secreted glycoproteins, such as cytokines, chemokines and antibodies, the sugar portion has been described to perform important functions. This is the case of the *N*-glycans attached to the Fc portion of IgG, that when sialylated send an inhibitory signal to the immune system (Kaneko *et al*., 2006; Anthony *et al*., 2011). Glycans are also central to lymphocyte development (Stanley and Okajima, 2010) and leucocyte homing (Lowe, 2003; Mitoma *et al*., 2007). Finally, an additional immunomodulatory pathway in which surface glycans have a major role is the lectin pathway (LP) for complement activation, which requires the mannose-binding lectin and ficolins, rather than the standard components (Matsushita, 2010) necessary for the activation of the classical and alternative pathways (AP). The identification of singular aspects related to glycan composition in kinetoplastids and their interaction with host lectins may unveil opportunities for drug design using agents that specifically bind to carbohydrate moieties important for parasite survival within the mammalian host.

## The nature of surface glycans in *Trypanosoma cruzi*

As in the case of other protozoan parasites of medical and veterinary relevance, the surface of *T. cruzi* is heavily glycosylated. The dense glycocalyx performs specific and significant functions such as protection against the host defence mechanisms and/or the interaction with changing environments (Noireau *et al*., 2009; Romano *et al*., 2012). The carbohydrate nature of the surface coat strongly depends on the life stage and differentiation involves unique changes in its composition (de Lederkremer and Agusti, 2009).

The most abundant components of the *T. cruzi* surface coat, especially in the epimastigote form, are glycosylphosphatidylinositol (GPI)-anchored glycoconjugates of varied nature (Ferguson, 1999). The structure of this coat has been described as a basal layer of glycoinositolphospholipids (GIPLs) and phospholipid (Previato *et al*., 1990; de Lederkremer *et al*., 1991; Carreira *et al*., 1996) from which other GPI-anchored molecules protrude (Previato *et al*., 2004). The major species are mucin-like proteins (Pereira-Chioccola *et al*., 2000; Buscaglia *et al*., 2006), which are heavily *O*-glycosylated, while the less abundant include *trans*-sialidase (TS) (Previato *et al*., 1985; Schenkman and Eichinger, 1993), mucin-associated proteins (MASPs) (dos Santos *et al*., 2012), Gp85 surface glycoproteins (Mattos *et al*., 2014), trypomastigote small surface antigen (TSSA) (Canepa *et al*., 2012) and Toll-T antigens (Quanquin *et al*., 1999). Recent studies support the idea that lipid-based domains, and particularly lipid rafts, are responsible for the fine organization of all these components (Mucci *et al*., 2017).

GIPL was the first glycoconjugate characterized in *T. cruzi* and can be found as a free entity or anchored to proteins. GIPLs were originally defined as lipopeptidophosphoglycans (LPPGs) because of the amino acids present in the early preparations (De Lederkremer *et al*., 1976). However, with the solution of their structure, it was established that these LPPGs are typical GPIs (Previato *et al*., 1990; de Lederkremer *et al*., 1991). The core of GIPLs is constituted by Man*α*(1,2)-Man*α*(1,6)-Man*α*(1,4)-GlcN*α*(1,6)-*myo-*inositol-PO_4_-lipid, in some cases with four mannose residues, where the lipid moiety is either 1-*O*-hexadecyl-2-*O*-palmitoyl glycerol or ceramide (McConville and Ferguson, 1993). Galactofuranose (Gal*f*) and aminoethylphosphonic acid are substituents that can be found attached to different positions of the main core, conferring a certain microheterogeneity to the oligosaccharide structure (McConville and Ferguson, 1993).

Mucins are the most abundant glycoproteins in the *T. cruzi* surface membrane. They are a complex and heterogeneous group of variable proteins constituted by a polypeptidic core of 50–200 amino acids, rich in serine and threonine residues many of which are *O*-glycosylated (Buscaglia *et al*., 2006). Mucins are *O*-glycosylated with *N*-acetylglucosamine (GlcNAc), which is rather unique since the glycosyltransferases that catalyse this transfer in other organisms usually use UDP-*N*-acetylgalactosamine as a precursor (Previato *et al*., 1995, 1998). The *O*-linked GlcNAc residues can be further elongated or remain unsubstituted. Galactose is present in all mucin oligosaccharide elongations in either the pyranosic (Gal*p*) or Gal*f* configuration (Acosta-Serrano *et al*., 2001). Terminal *β*-Gal*p* residues can be further branched with sialic acid acquired from the host through TSs present on the membrane surface (Previato *et al*., 1994; Serrano *et al*., 1995).

TSs are another group of GPI-anchored proteins found on the surface of *T. cruzi*, and their activity allows the parasite to bypass its lack of *de novo* synthesis of sialic acid, that is instead salvaged from the host (Previato *et al*., 1985). TSs catalyse the transfer of sialic acid from an *α*(2,3)-linkage in the donor to a terminal *β*-Gal*p* acceptor of the parasite mucins (Schenkman *et al*., 1993). It has also been shown that *T. cruzi* TS (TcTS) can efficiently transfer *α*(2,3)-linked *N*-glycolylneuraminic acid (Neu5Gc) to terminal *β*-Gal groups (Agusti *et al*., 2007; Schroven *et al*., 2007). This specific activity of TcTS is unique because of several aspects. First, TcTSs, unlike mammalian TSs, do not use cytidine monophospho (CMP)-sialic acid as the monosaccharide donor. Additionally, they appear to be located on the parasite surface and not in the Golgi apparatus, which is where they carry out their normal function in other organisms. Finally, unlike conventional sialidases, TcTSs are more efficient in transferring terminal sialic acids between glycoconjugates rather than hydrolysing them. Recent studies have also shown that sialylated mucins are present in membrane lipid-rafts far away from TS and that the sialylation process is performed by microvesicles associated with active TcTS (Lantos *et al*., 2016).

Other GPI-anchored proteins are the Gp85 surface glycoproteins, TSSAs and MASPs. Gp85 glycoproteins are usually included in the TS superfamily as TS-like proteins yet they lack TS activity (Buscaglia *et al*., 2006) and appear to be involved in host–parasite interactions (Alves and Colli, 2008). TSSAs are polymorphic mucin-like molecules with a conserved hydrophobic C-terminus compatible with the GPI-anchoring signal, and a variable central region responsible for their antigenicity (Di Noia *et al*., 2002). Finally, MASPs are GPI-anchored proteins that have been found predominantly in the proteome of trypomastigotes (Atwood *et al*., 2005). Like mucins, they contain highly conserved N- and C-terminal domains plus a variable central region (Bartholomeu *et al*., 2009) yet they appear to be *N*-glycosylated (Atwood *et al*., 2006).

### Glycans and immunomodulation during *T. cruzi* infection

**Fig. 1.**
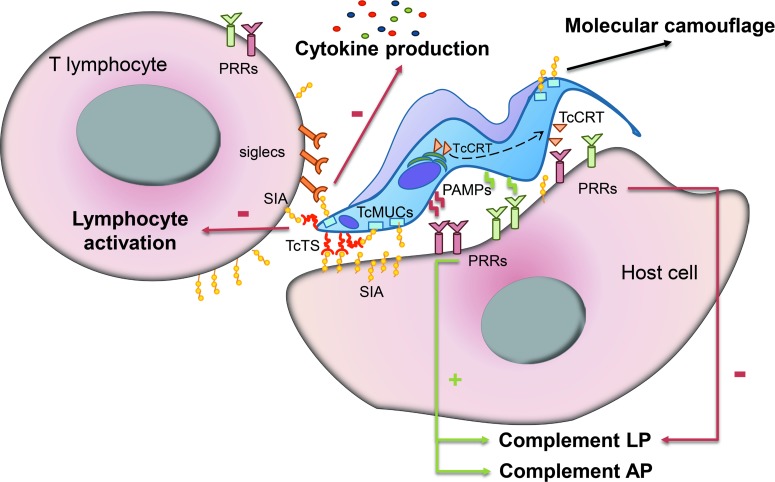
Scheme of the interplay between *T. cruzi* surface glycans and mammalian host cells. Upon infection, surface glycans within PAMPs can interact with host cell (i.e. myeloid and dendritic cells) PRRs and lead to the activation of the complement LP and AP. TcCRT translocates from the endoplasmic reticulum to the surface membrane in the zone of flagellum emergence and interacts with PRRs interfering in the normal activation of the complement LP and AP. Sialic acid (SIA) is transferred from the host cell membrane to parasite surface proteins such as mucins (TcMUC), conferring this way a molecular camouflage that hinders an effective immune response. The transfer of SIA is catalysed by TcTS and leads to an inhibition of the activation of T lymphocytes. In addition, sialylated mucins may interact with siglecs expressed on the surface of T cells and inhibit cytokine production.

The complement is the first line of defence of the innate immune system against invading microbes. *Trypanosoma cruzi* invasion generates an immediate immune response due to the interaction of the parasite with complement molecules. It has been shown that the complement can be activated by all *T. cruzi* forms: amastigote (Iida *et al*., 1989), epimastigote (Nogueira *et al*., 1975) and trypomastigote (Kipnis *et al*., 1985), but only the non-infective epimastigotes are susceptible to complement lysis. During the first seconds after *T. cruzi* infection, signal glycoproteins on the parasite surface can interact with host PRRs such as mannose-binding lectins and ficolins and lead to the activation of the LP and AP (Fig. 1) (Cestari *et al*., 2013). However, *T. cruzi* parasites can undertake a series of strategies to escape the effects of both innate and adaptive immunity. There are at least three different mechanisms of complement system evasion by *T. cruzi*. One of such mechanisms is the translocation of calreticulin (TcCRT), a calcium binding protein normally expressed in the endoplasmic reticulum, to the surface membrane on the flagellar portion of the parasite (Ferreira *et al*., 2004*a*, 2004*b*; Gonzalez *et al*., 2015). This translocation allows TcCRT to interact with mannose-binding lectins and ficolins and this way interfere with the normal activation of the LP and classical pathway and enhance the rate of the internalization of parasites by host cells (Fig. 1) (Gonzalez *et al*., 2015). Another escape mechanism from the innate immune response is the release of plasma membrane microvesicles by *T. cruzi* parasites. Extracellular vesicles contain several signal factors including glycoproteins and enzymes involved in carbohydrate metabolism, which also interfere with the LP and classical pathway activation (Geiger *et al*., 2010; Ramirez *et al*., 2017).

One of the most important carbohydrates interfering with the immune response against *T. cruzi* infection is sialic acid. *Trypanosoma cruzi* transfers sialic acid from the host to its own surface glycoproteins creating this way a perfect molecular camouflage that hinders an effective immune response (Fig. 1) (Argibay *et al*., 2002; Gao *et al*., 2002; Freire-de-Lima *et al*., 2010). In addition, sialylated mucins may interact with siglecs expressed on the T cell surface and inhibit clonal expansion and cytokine production by CD4^+^ lymphocytes (Nunes *et al*., 2013). On the other hand, TcTS interferes with the activation of T lymphocytes (Fig. 1) (Pennock *et al*., 2013). This latter process involves the loss of sialic acid residues from *O*-linked oligosaccharides and the exposure of free Gal*β*(1,3) residues (Galvan *et al*., 1998; Priatel *et al*., 2000).

### Host cell invasion

*Trypanosoma cruzi* has a quite complex life cycle that involves an obligate intracellular stage for parasite duplication. Cell invasion involves a strict and complex interaction between the parasite and the host cell. The first step of this process is the adhesion of the parasite to the target cell which involves the recognition of molecules present on the surface of both parasite and host cells. Several molecules of *T. cruzi* surface are involved, among them glycoproteins of the Gp85/TS family, and mucins are of greatest interest. *β*-Gal residues on surface glycoproteins have been suggested to mediate parasite attachment and entry in dendritic (Vray *et al*., 2004) and smooth muscle cells (Kleshchenko *et al*., 2004; Vray *et al*., 2004). In addition, cruzipain, a major cysteine peptidase has also a role in immune evasion, host cell invasion and intracellular development. After the binding and recognition of the parasite by the host cell surface, *T. cruzi* is internalized by two possible mechanisms: phagocytosis (Vieira *et al*., 2002) and endocytosis (Schenkman and Mortara, 1992). Once inside the host cell, parasites are confined in the parasitophorous vacuole, a membrane structure that protects them from lysosome attack while replicating. Probably one of the most important events of host cell invasion is the ‘escape’ from the parasitophorous vacuole. Also in this process surface glycoproteins have a major function (Andrews and Whitlow, 1989; Stecconi-Silva *et al*., 2003).

## Surface glycans in *Trypanosoma brucei*

The *T. brucei* surface coat exhibits a dense layer of GPI-anchored glycoproteins, such as the variant surface glycoproteins (VSGs) or procyclin found in the bloodstream or procyclic forms of the parasite, respectively. In a minor amount, other glycosylated proteins are expressed in the surface membrane, such as the trans-membrane invariant surface glycoproteins (ISGs) (Ziegelbauer and Overath, 1992; Ziegelbauer *et al*., 1992), the transferrin receptor (TfR) (Grab *et al*., 1993) and the haptoglobin–haemoglobin receptor (Vanhollebeke *et al*., 2008) which are both GPI-anchored and located in the flagellar pocket. In addition, it has been reported that epimastigote forms found in the salivary glands of the tsetse fly present a stage-specific coat of a GPI-anchored protein named bloodstream stage alanine-rich protein or Brucei alanine-rich protein (BARP) (Nolan *et al*., 2000; Urwyler *et al*., 2007). The differentiation of epimastigotes to metacyclic trypomastigote forms is associated with the loss of BARP and to the expression of a new coat of metacyclic VSGs (Tetley *et al*., 1987; Ginger *et al*., 2002) and of a small family of metacyclic invariant surface glycoproteins which protrude and remain accessible for antibody recognition (Casas-Sánchez *et al*., 2018). All these glycoproteins are mainly *N*-glycosylated with different structures containing oligomannose, paucimannose and complex-type glycans (Bangs *et al*., 1988; Zamze *et al*., 1990; Strang *et al*., 1993; Treumann *et al*., 1997; Mehlert *et al*., 1998*a*, 1998*b*, 2012; Acosta-Serrano *et al*., 2004).

Specifically, VSGs are homodimers susceptible to *N*-glycosylation with one, two or three *N*-linked oligosaccharides depending on the VSG class. Thus, in Class 1 VSGs only one asparagine is modified with triantennary oligomannose structures (Man_9–5_GlcNAc_2_); Class 2 VSGs have two *N*-glycosylation sites, one of them is occupied with oligomannose structures and the other with structure type Man_4–3_GlcNAc_2_ and biantennary complex glycans and Class 3 VSGs are modified with a combination of oligomannose and complex biantennary glycans (Zamze *et al*., 1990, 1991; Mehlert *et al*., 1998*b*). Recently, it has been reported that VSGs can also be *O*-glycosylated (Pinger *et al*., 2018).

TfR is a heterodimeric protein expressed in the bloodstream form that is encoded by expression site-associated genes (ESAGs) 6 and 7 exhibiting eight *N*-glycosylation sequons. Both ESAG6 and ESAG7 are heterogeneously *N*-glycosylated with paucimannose and oligomannose moieties such as VSGs but not with complex *N*-glycans (Mehlert *et al*., 2012). In contrast to the glycoproteins described above, the *N*-glycosylation profile of the ISGs has not been characterized so far.

On the other hand, the GPI structure built-up by NH_2_CH_2_CH_2_-PO_4_H-6-Man*α*(1,2)-Man*α*(1,6)-Man*α*(1,4)-GlcN*α*(1,6)-*myo*-inositol-1-PO_4_H-3(*sn*-1,2-dimyristoylglycerol) by which the aforementioned glycoproteins, except for ISGs, are anchored to the surface membrane is further modified by *N*-glycosylation (Holder, 1985; Ferguson *et al*., 1988; Redman *et al*., 1994).

### Glycans and interaction of *T. brucei* with the mammalian host

*Trypanosoma brucei* parasites dwelling in the mammalian bloodstream are exposed to innate and adaptive responses by the immune system for which they have developed sophisticated evasion strategies. An essential mechanism for effective immune evasion is the antigenic variation of VSGs whereby parasites switch to a new, immunologically distinct VSG, selected from among a huge collection of silent VSG genes. At the initial stages of the humoral immune response, when antibody levels are still low, the VSG–antibody complexes are rapidly internalized at the flagellar pocket by clathrin-dependent endocytosis, to be further dissociated in isolated VSG which is recycled to the surface, and the Ig that is directed to the lysosome to be proteolysed (O'Beirne *et al*., 1998; Pal *et al*., 2003; Engstler *et al*., 2004; Overath and Engstler, 2004). Antibody internalization becomes insufficient as the titre increases, and the complement system, mediated by specific antibodies against the predominant form of VSG, promotes efficient opsonization and lysis of parasites except for those expressing the new VSG that will spread again the infection, as they are able to escape the adaptive immune response. As the infection progresses, slender proliferative bloodstream parasites differentiate into a stumpy non-proliferative form that plays an important role in different ways. It contributes to avoid massive parasitaemia and premature host death, allows for pre-adaptation to the tsetse fly and by reducing the VSG repertoire expression it restricts antibody generation by the host, thus extending the functionality of antigenic variation (MacGregor *et al*., 2011; Matthews, 2015).

**Fig. 2.**
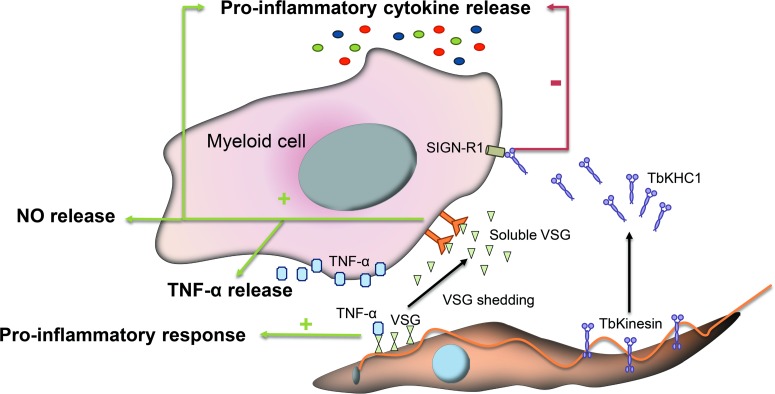
Immunomodulatory events mediated by glycans during infection with *T. brucei*. VSGs interact with host immune cells and act as immunomodulatory factors. The conserved VSGs chitobiose-oligomannose moiety of VSGs binds to TNF-*α*, a cytokine with lectin-like properties and induces a pro-inflammatory response. Likewise, during differentiation to stumpy forms a VSG shedding process takes place allowing for the release of soluble VSG portions into the bloodstream of the mammalian host. These fragmented VSGs containing glycosylinositolphosphate induce myeloid cell activation and thereby the expression of pro-inflammatory cytokines and the release of NO and TNF-*α*. Other parasite-released factors interfere with the pro-inflammatory response such as the Kinesin Heavy Chain 1 (TbKHC1), which binds to the mannose-specific Intercellular Adhesion Molecule-3-Grabbing Nonintegrin-Related 1 (SIGN-R1) receptor and inhibits the host pro-inflammatory response.

While trypanosomes mainly rely on antigenic variation to circumvent immune detection, VSG glycosylation modulates host–parasite interactions, contributing to the formation of an efficient surface barrier, with increased antigenic variability and protective properties (Blum *et al*., 1993). Supporting this notion, it has been shown that *O*-glycosylation of VSGs confers the parasite additional surface heterogeneity, impairs the functionality of the host immune response and enhances parasite virulence (Pinger *et al*., 2018). Furthermore, specific carbohydrate branches at the trypanosome surface are involved in the process of binding and uptake of host macromolecules. The conserved VSGs chitobiose-oligomannose (GlcNAc_2_-Man_5–9_) moieties have been proposed to act as ligands for TNF-*α*, a cytokine with lectin-like properties inducing a pro-inflammatory response and parasite lysis (Fig. 2) (Magez *et al*., 2001) although the direct induction of trypanolysis by TNF-*α* has been recently questioned (Vanwalleghem *et al*., 2017).

VSGs also act as immunomodulatory factors involved in the production of TNF-*α* and nitric oxide (NO). During the differentiation process, slender trypanosomes suffer a VSG shedding process releasing to the bloodstream of the mammalian host soluble VSG portions from the membrane (Gruszynski *et al*., 2003). These fragmented VSGs containing glycosylinositolphosphate induce myeloid cell activation and thereby the expression of pro-inflammatory cytokines (Fig. 2) (Leppert *et al*., 2007). This process is amplified by T cell activation and IFN-*γ* release, which promotes macrophages to achieve a whole activated/M1 polarization and consequently increases TNF-*α* and NO secretion to control the infection (Stijlemans *et al*., 2016).

Other parasite-released factors interfere with the pro-inflammatory response of activated macrophages thus contributing to parasite infection. For instance, Kinesin Heavy Chain 1 (TbKHC1) is released in the blood by the parasite and interacts with the mannose-specific intercellular adhesion molecule-3-grabbing nonintegrin-related 1 (SIGN-R1) receptor, a C-type lectin expressed in the surface of immune cells to inhibit the host pro-inflammatory response and at the same time stimulates the production by the host of essential trypanosomal nutrients (Fig. 2). TbKHC1 reduces the conversion of L-arginine into NO by inducing arginase-1 activity *via* IL-10 of macrophages/myeloid cells. The modulation of arginase activity promotes the formation of L-ornithine and consequently of polyamines required for trypanosome growth (De Muylder *et al*., 2013).

Other glycoproteins that play major roles in host–parasite interaction are the trypanosome-derived lymphocyte-triggering factor, which is secreted by *T. brucei* parasites promoting early IFN-*γ* production by CD8^+^ T lymphocytes (Vaidya *et al*., 1997), the GPI-phospholipase C, a bloodstream stage-specific enzyme that is concentrated in the flagellar membrane and participates in VSG shedding during differentiation of bloodstream forms to procyclic forms (Grandgenett *et al*., 2007) and the TfR, which is located in the flagellar pocket and is involved in providing iron to the parasite (Steverding *et al*., 1994). The *N*-glycosylation of both TfR protein subunits has been proposed to provide a spatial localization in the plasma membrane together with the VSG coat that allows transferrin binding without significant exposure to the immune system (Mehlert *et al*., 2012).

## Surface glycans in *Leishmania*

In the case of *Leishmania*, the surface coat is covered by a dense external glycocalyx harbouring different glycoconjugates with an important role in the parasite–host interaction. Its nature varies between species and different forms of the parasite during the life cycle. Promastigote cells contain a series of glycoconjugates, which include GPI-anchored proteins such as the metalloprotease leishmanolysin/GP63, the parasite surface antigen-2 complex (PSA-2/GP46) and the mucin-like proteophosphoglycan (PPG), a complex GPI-anchored lipophosphoglycan (LPG) and low molecular weight GIPLs which are not attached to either proteins or polysaccharides. *Leishmania* also secretes protein-linked phosphoglycans, such as the secreted proteophosphoglycan (sPPG) and secreted acid phosphatase (Sacks and Kamhawi, 2001).

LPG is the main cell-surface glycoconjugate in promastigotes covering the whole parasite including the flagellum. It is comprised a 1-*O*-alkyl-2-*lyso*-phosphatidyl(*myo*)inositol lipid anchor with a heptasaccharide glycan core, Gal*pβ*(1,6)- Gal*pβ*(1,3)-Gal*f*α(1,3)(Glc*β*(1)-PO_4_-(6))-Man*β*(1,3)-Man*β*(1,4)-GlcN, which is joined to a long polyglycosyl phosphate (PG) consisting of repeating units of a disaccharide and a phosphate (Gal*β*(1,4)-Man*α*(1)-PO_4_) and terminated by an oligosaccharide cap structure consisting of Man*α*(1,2)-Man*α*(1) or as Gal*β*(1,4)(Man*α*(1,2))-Man*α*(1). The PG units appear to be modified by carbohydrate chains that differ markedly between species and stage (McConville *et al*., 1995). In amastigotes, LPG expression is highly downregulated (Turco and Sacks, 1991).

PPG is the second major phosphoglycan but, unlike LPG, it contains a polypeptide backbone rich in serines to which phosphoglycans are linked *via* phosphodiester bonds. The PG molecules consist of three differently branched phosphodisaccharides that end with a neutral capping structure (Ilg, 2000).

GP63 is the most abundant surface glycoprotein expressed on the *Leishmania* promastigote cell membrane. GP63 is *N*-glycosylated with paucimannose structures such as Man_6_GlcNAc_2_ and GlcMan_6_GlcNAc_2_ (Funk *et al*., 1997), and Man_3_GlcN structure in the GPI-anchor (Cabezas *et al*., 2015). It is a zinc-dependent protease with a wide range of substrates including casein, haemoglobin, fibrinogen, etc. (Yao *et al*., 2003).

Differentiation to the amastigote form involves the thinning of the glycocalyx; in addition to LPG, the levels of GP63 are also dramatically decreased and GIPLs become the major surface glycoconjugate in this form (McConville and Blackwell, 1991; Schneider *et al*., 1992; Winter *et al*., 1994). GIPLs are composed of the Man*α*(1,4)-GlcN*α*(1,6)-*myo*-inositol unit, which is substituted with either high mannose (type-1) or Gal*p*-Gal*f* (type-2) structures or with both forming a hybrid glycoside (McConville and Ferguson, 1993; Cabezas *et al*., 2015). Like in *T. cruzi*, *Leishmania* also presents its cell surface decorated with sialic acid-bearing glycoconjugates. *Leishmania donovani* promastigotes exhibit 9-*O*-acetylated sialic acid and distinct 9-*O*-acetylated sialoglycoproteins while the amastigote form harbours an unusual derivative of sialic acid, Neu5Gc, absent in promastigotes (Ghoshal and Mandal, 2011).

### Role of *Leishmania* surface glycans in immunomodulation and parasite–host cell interactions

**Fig. 3.**
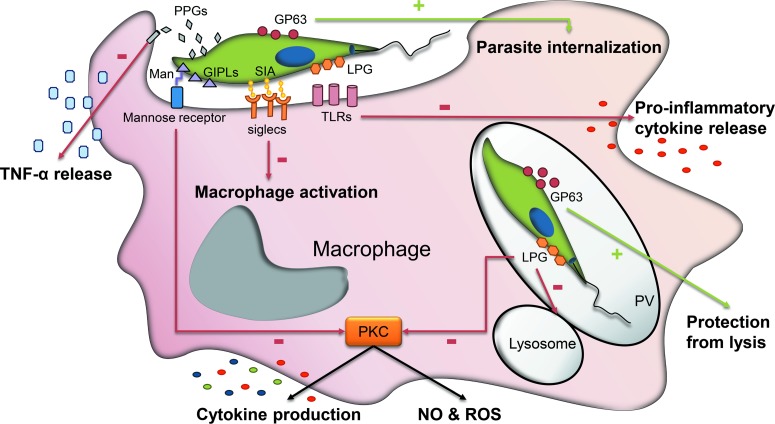
Schematic representation of the major parasite–macrophage interactions mediated by surface glycans in *Leishmania*. The major glycoconjugates involved in the parasite–macrophage interplay are indicated: PPG, GPI-anchored LPG GIPLs and the metalloprotease GP63. After infection, promastigote LPG triggers TLR signalling and interferes with pro-inflammatory and signalling pathways. Once inside the macrophage, LPG delays the fusion of the parasitophorous vacuole with lysosomes and inhibits protein kinase C and therefore, the production of cytokines and the oxidative and nitrosative stress response. Likewise, mannose-terminating GIPLs interact with mannose receptors on the macrophage surface and inhibit PKC activity. sPPGs impair important macrophage functions such as the release of TNF-*α*. Finally, GP63 is an important virulence factor which, among other functions, promotes *Leishmania* internalization and facilitates escape from lysis by the complement pathway.

The presence of a cell surface glycocalyx has a critical role in host–parasite interactions and infectivity thanks to an array of well-defined epitopes of branched *N*-glycans that act as ligands for receptors on cells of the insect or the vertebrate host. In *Leishmania* promastigotes, the dense glycocalyx formed by LPG performs a number of functions for parasite survival within the insect and for macrophage infection within the mammalian host. LPGs confer physical protection against digestive hydrolytic enzymes of the sandfly and are involved in the attachment to the gut epithelium and migration of metacyclic parasites to the mouthparts of the insect (Ilg, 2000; Sacks and Kamhawi, 2001). In the blood stream, LPG prevents lysis by complement proteins and serves as a ligand for attachment and receptor-mediated phagocytosis by the macrophage. LPG triggers TLR signalling and interferes with pro-inflammatory and signalling pathways in host cells (Fig. 3) (Becker *et al*., 2003; Rojas-Bernabe *et al*., 2014). Once inside the macrophage, LPG delays the fusion of the parasitophorous vacuole with lysosomes and inhibits protein kinase C and the production of cytokines related to the microbicidal oxidative and nitrosative stress response (Fig. 3) (Descoteaux and Turco, 1993; Kavoosi *et al*., 2009; Franco *et al*., 2012).

Besides LPG, other glycoconjugates such as GIPLs and PPGs are involved in the first stages of macrophage infection (McConville and Blackwell, 1991; Piani *et al*., 1999). Mannose-terminating GIPLs interact with mannose receptors on the macrophage surface (Blackwell *et al*., 1985) and modulate many macrophage functions such as PKC activity (Chawla and Vishwakarma, 2003), cytokine production, release of NO and differentially activate MAPK (Fig. 3) (Assis *et al*., 2012). While the implication of GIPLs in *Leishmania*–macrophages interaction is well established, their role in intramacrophage development is still unclear. On the other hand, PPGs play important biological roles in the establishment of *Leishmania* infection and virulence (Capul *et al*., 2007; Gaur *et al*., 2009; Olivier *et al*., 2012). Filamentous PPGs are found in the promastigote secretory gel, a viscous mucin-like material which accumulates in sandfly gut and mouthparts and improves *Leishmania* transmission by promoting multiple insect bites and increasing the number of parasites per bite (Rogers *et al*., 2004; Rogers and Bates, 2007). PPGs regurgitated by *Leishmania*-infected sandflies favour macrophage recruitment to the bite site and target the L-arginine metabolism of host macrophages to promote establishment of the infection (Rogers *et al*., 2009). In a murine model, sPPG has been shown to inhibit TNF-*α* release to facilitate the establishment of the infection (Piani *et al*., 1999).

GPI-anchored glycoproteins that exhibit pivotal functions in the parasite–mammalian host interplay in *Leishmania* are GP63 and PSA-2. Metalloprotease GP63 is abundant in promastigotes but expressed to a lesser extent in amastigotes (Schneider *et al*., 1992). GP63 is an important virulence factor which modulates a wide range of host cell signalling pathways that regulate macrophage anti-microbial and inflammatory functions. GP63 facilitates parasite escape from lysis by the complement pathway and the movement through the extracellular matrix, favours promastigote internalization into macrophages, inhibits natural killer cell responses, promotes resistance to antimicrobial peptides and seems to play a key role in protecting intracellular parasites from the hostile environment of macrophages (Fig. 3) (Olivier *et al*., 2012; Yao *et al*., 2003, 2010). Likewise, the PSA-2 protein is involved in the binding and invasion of parasites on macrophages and resistance to complement lysis (Kedzierski *et al*., 2004; Lincoln *et al*., 2004).

Another type of interaction with host cells by which parasites can establish successful infection takes place through sialic acid-siglec binding. *Leishmania* utilizes sialic acids to bind these membrane-bound receptors present in the haematopoetic cell lineages promoting parasite entry within macrophages, NO-resistance, host immunomodulation and strain virulence (Fig. 3) (Ghoshal and Mandal, 2011; Roy and Mandal, 2016).

## Carbohydrate-binding agents as antimicrobials

Several studies support the therapeutic potential of carbohydrate-binding agents (CBAs). Lectins are CBAs of peptidic nature that specifically bind diverse carbohydrate structures. By acting as recognition and adhesion molecules and as signal transducers they perform a wide variety of physiological functions. Cell membrane proteins and lipids in many pathogens exhibit specific glycosylation patterns different from the mammalian host and are potential binding sites for lectins of selected specificity. Thus, lectins naturally occurring in plants, microbes, animals and humans exhibit antimicrobial activity (Petrova *et al*., 2016; Zhang and Gallo, 2016) through the interaction with complex carbohydrates on microbial surfaces and there is growing interest in their applicability due to the possible interference with host cell–pathogen interactions and disease development (Breitenbach Barroso Coelho *et al*., 2018). Decreased capacity of invasion and infection, inhibition of proliferation and impairment of pathogen cell adhesion and migration has been reported to occur upon incubation with lectins from different sources (da Silva *et al*., 2019; Hasan and Ozeki, 2019; Li *et al*., 2019). In addition, lectins have been acknowledged as promising potential carrier molecules for directed drug delivery (Žurga *et al*., 2017) since by binding to membrane glycan moieties, they can elicit cell internalization of molecules of therapeutic interest.

Indeed with regards to their anti-infective potential, multiple studies have demonstrated the enormous antiviral capacities of CBAs (Francois and Balzarini, 2012; Gondim *et al*., 2019). The infectivity of several viruses requires surface glycoproteins and interference with host cell recognition has been the basis of the antiviral activity of lectins that specifically bind mannose-rich surface glycans (Dey *et al*., 2000; Hoorelbeke *et al*., 2010). Prolonged exposure to CBAs resulted in defects in the glycosylation status of surface glycoproteins giving rise to defective binding and increased exposure of underlying epitopes to the host immune response adding this way a new feature to their mode of action (Balzarini *et al*., 2005). In addition, non-peptidic CBAs have been successfully used in the treatment of fungal infections both *in vitro* and *in vivo* supporting the possible employment of this class of compounds in a clinical setting (Tomita *et al*., 1990; Walsh and Giri, 1997).

## Therapeutic opportunities in kinetoplastids

In the case of kinetoplastid diseases, treatment often suffers from toxicity, side-effects and limited efficacy. New entities with novel modes of action are therefore needed to address the increasing demand for novel medicines. Despite extensive screening and *in vitro* and *in vivo* studies, only very few compounds have advanced to clinical trials. Taking into account the importance of protein glycosylation, the unique character of cell surface glycans during the infective stages of parasitic protozoa opens exciting possibilities for the use of CBAs as antiparasitics. A mode of action can be foreseen where these agents would act directly exerting toxicity by binding to the cell surface and inducing parasite lysis and/or additionally by preventing pathogen infection in the host by impairing crucial interactions involved in the attenuation of the immune response or parasite uptake. The direct cytotoxic activity upon incubation with CBAs has been demonstrated in *T. brucei* bloodstream forms where peptidic agents such as the amaryllis lectin *Hippeastrum* hybrid (HHA) and the stinging nettle lectin (UDA) from *Urtica dioica* perturb endocytosis (Castillo-Acosta *et al*., 2013, 2015). Likewise, a specific block in endocytosis was observed after exposure of *T. cruzi* to a Poly-LAcNAc binding lectin (Brosson *et al*., 2016). However, while many plant peptidic CBAs exist with a wide range of glycan specificities, including mannose, galactose, glucose, fucose, sialic acid, GlcNAc and GalNAc oligomers, the approach involves major challenges. The toxicity of many peptidic lectins precludes their use as drugs and the identification of CBAs that exhibit selectivity towards parasite glycans and low toxicity towards mammalian cells are required.

Previous studies on the utility of CBAs in kinetoplastid diseases are limited. Certain plant lectins have demonstrated utility as adjuvants when studying the mouse humoral immune response to *T. cruzi* (Albuquerque *et al*., 1999). The cramoll 1,4 lectin, is a protein that recognizes and interacts with specific glycans on the cell surface inducing mitogenic activity (Maciel *et al*., 2004) which in *T. cruzi*, induces changes in plasma membrane permeability, production of reactive oxygen species and defects in mitochondrial function (Fernandes *et al*., 2010). A protective effect of lectin administration in *Leishmania* infections has also been documented. Thus, lectins such as the ConBr from *Canavalia brasiliensis* and KM+ from *Artocarpus integrifolia* induce IFN-*γ* and IL-12 p40 production promoting a reversal of the Th2 cytokine pattern to Th1 pattern in BALB/c mice infected with *Leishmania amazonensis* and *Leishmania major*, respectively (Barral-Netto *et al*., 1996; Panunto-Castelo *et al*., 2001). Pretreatment of murine inflammatory peritoneal macrophages with a D-galactose-binding lectin from *Synadenium carinatum* latex (ScLL) reduced by 65.5% the association index of macrophages and *L. amazonensis* promastigotes (Afonso-Cardoso *et al*., 2011). ScLL also reduced the growth of *L. amazonensis* amastigote intracellular forms, showing no *in vitro* cytotoxic effects in mammalian host cells (Afonso-Cardoso *et al*., 2011).

Remarkably, evidence has been recently presented showing that the use of CBAs could involve a completely novel approach to chemotherapy of protozoan-infectious diseases in the case of sleeping sickness. Thus products from natural sources of both peptidic and non-peptidic nature have demonstrated their antitrypanosomal potential in the case of *T. brucei* both *in vitro* and *in vivo* (Castillo-Acosta *et al*., 2013, 2016). Certain *α*(1,3)-*α*(1,6)Man-specific peptidic CBAs such as the HHA or the *α*(1,3)Man-specific snowdrop lectin from *Galanthus nivalis* (GNA) inhibit growth of bloodstream forms of *T. brucei* while exhibiting very low toxicity against mammalian cells *in vitro* (Castillo-Acosta *et al*., 2013) although as peptides their therapeutic potential was limited. In the case of non-peptidic glycan binding agents, the identification of low molecular weight, non-toxic compounds to date has been restricted mostly to the natural products from *Actinomycetes* namely pradimicin A (Walsh and Giri, 1997) and benanomicin A (Watanabe *et al*., 1996) and their synthetic analogues although several groups have aimed at the synthesis and characterization of synthetic CBAs that bind specific oligosaccharide structures (Striegler and Dittel, 2003; Mazik *et al*., 2005). Indeed treatment with the non-peptidic CBA pradimicin S procured parasitological cure in mouse models of acute sleeping sickness with no evidence of toxicity or side-effects further supporting the potential of the approach (Castillo-Acosta *et al*., 2016). Pradimicin A exhibits *α*(1,2)Man specificity and binds tightly to the parasite VSGs presumably through specific interactions with oligomannose surface glycans that are highly abundant in the bloodstream form of the parasite. Binding of pradimicin to bloodstream form trypanosomes induced defects in endocytosis and parasite lysis. Interestingly induction of resistance to pradimicin A *in vitro* resulted in parasites with defective glycosylation and a reduction in the content of mannose-rich glycans that exhibited reduced infectivity thus clearly supporting the proposed mechanism of action (Castillo-Acosta *et al*., 2016). Binding to mannose-rich surface glycoproteins was also the basis for the potent activity of pradimicins against HIV by the interaction with the heavily mannosylated surface glycoprotein gp120 (Balzarini *et al*., 2005).

When understanding the antiprotozoal activity of CBAs, as previously mentioned, the complement system, mediated by specific antibodies against VSGs, allows for efficient opsonization and lysis of parasites. Considering the capability of CBAs to interact with *T. brucei* membrane glycoproteins and in particular VSGs, we hypothesize that CBAs could act as opsonins *per se* and therefore increase phagocytosis by macrophages. In addition, it is possible that the endocytosis block mediated by CBAs may also interfere with VSG recycling leading to a reduced clearance of surface coat antibodies and further promoting the opsonization process. On the other hand, CBAs may bind to specific carbohydrate branches at the trypanosome surface that are crucially involved in the process of binding of host macromolecules. Thus in *T. brucei* transferrin binding by the TfR could also be compromised (Mehlert *et al*., 2012). Furthermore, TbKHC1 interacts with the mannose-specific SIGN-R1 receptor and inhibits the pro-inflammatory response of the host (De Muylder *et al*., 2013). Again it is possible that CBAs interfere with these or other events that may be related to its mode of action *in vivo*.

In the case of *T. cruzi*, the core of GIPLs, a major surface constituent, is made up by Man*α*(1,2)-Man*α*(1,6)-Man*α*(1,4)-GlcN*α*(1,6)-*myo-*inositol-PO_4_-lipid (McConville and Ferguson, 1993) while galactose can be found attached to different positions. As previously mentioned TcCRT, which interacts with host PRRs mannose-binding lectins, increases host cell parasite internalization. Perturbation of this process through the use of mannose binding CBAs could reduce infection of new cells. The abundant mucins contain galactose in their oligosaccharide elongations and are *O*-glycosylated with GlcNAc. In addition, the exposure of free *β*(1,3)Gal residues and *β*-Gal residues has been suggested to mediate parasite attachment and entry in dendritic cells. Indeed, human galectin-3, a member of the lectin family with affinity to *β*-Gal and derivatives, plays a pivotal role in controlling *T. cruzi* infection. It has been recently proposed that galectin-3 deficiency during *T. cruzi* experimental infection resulted in increased *in vivo* systemic parasitaemia, and reduced leucocyte recruitment (da Silva *et al*., 2017). Since galactose-specific lectins are available, their possible interaction with infection mechanisms in the case of *T. cruzi* warrants investigation of the potential of this class of CBAs in the case of Chagas disease.

Finally, the use of treatment with CBAs in the case of infections caused by *Leishmania* spp. should also be further considered. *Leishmania* can target several macrophage membrane-bound receptors to subvert the inflammatory response. Mannose-terminating GIPLs interact with mannose receptors on the macrophage surface (Blackwell *et al*., 1985) modulating macrophage functions such as PKC activity (Chawla and Vishwakarma, 2003), cytokine levels and the production of NO. Sialic acids on the parasite surface interact with siglec receptors on macrophages to diminish the immune response (Roy and Mandal, 2016). Additionally, of interest is the observation that TLR-2 is involved in parasite survival in macrophages upon activation by LPG and interactions between LPG and TLR-2 reduce anti-leishmanial responses *via* cytokine-mediated decrease of TLR-9 expression (Srivastava *et al*., 2013). All these important mechanisms of parasite survival may be perturbed upon treatment with mannose-specific CBAs.

## Concluding remarks

As highlighted in the present review, kinetoplastids interact with mammalian host cells by recognizing specific glycan ligands. Parasite surface carbohydrates are involved in parasite attachment and entry as well as in the modulation of the immune response and the progression of infection. Based on observations with lectins and non-peptidic pradimicins in the case of sleeping sickness, the use of CBAs emerges as a promising antitrypanosomal strategy. Specificity of the interactions and the unique structure of kinetoplastid surface sugars may provide a basis for future drug design. Many bioactive natural carbohydrate-binding compounds are present in nature that often exhibit exquisite specificity for binding to carbohydrates, particularly carbohydrate sequences that occur on the surface of living cells. These molecules have the potential for treatment of kinetoplastid diseases. While specificity and toxicity may constitute important issues, the possibility of identifying agents that can be used to block the attachment of the parasite to cell surfaces (or interfere with the subversion of the immune response), and thus prevent or suppress infection is appealing. Future identification of new CBAs with improved pharmacological profiles and reduced side-effects may provide novel avenues for the exploitation of this innovative concept.
